# Early life adversity impacts alterations in brain structure and food addiction in individuals with high BMI

**DOI:** 10.1038/s41598-024-63414-z

**Published:** 2024-06-07

**Authors:** Soumya Ravichandran, Riya Sood, Isha Das, Tien Dong, Johnny D. Figueroa, Jennifer Yang, Nicholas Finger, Allison Vaughan, Priten Vora, Katie Selvaraj, Jennifer S. Labus, Arpana Gupta

**Affiliations:** 1 Vatche and Tamar Manoukian Division of Digestive Diseases, David Geffen School of Medicine, University of California, Los Angeles, USA; 2 Goodman Luskin Microbiome Center, University of California, Los Angeles, USA; 3 G. Oppenheimer Center for Neurobiology of Stress and Resilience, The Obesity and Ingestive Behavior Program, Vatche and Tamar Manoukian Division of Digestive Diseases, David Geffen School of Medicine, University of California, 10833 Le Conte Avenue, Center for Health Sciences 42-210, Los Angeles, CA 90095 USA; 4 David Geffen School of Medicine, University of California, Los Angeles, USA; 5 UC San Diego School of Medicine, University of California, San Diego, USA; 6 Center for Health Disparities and Molecular Medicine, Department of Basic Sciences, Loma Linda University School of Medicine, Loma Linda, USA

**Keywords:** Obesity, Food addition, Early life adversity, Resilience, Reward networks, Reward, Obesity, Stress and resilience

## Abstract

Obesity and food addiction are associated with distinct brain signatures related to reward processing, and early life adversity (ELA) also increases alterations in these same reward regions. However, the neural mechanisms underlying the effect of early life adversity on food addiction are unknown. Therefore, the aim of this study was to examine the interactions between ELA, food addiction, and brain morphometry in individuals with obesity. 114 participants with high body mass index (BMI) underwent structural MRIs, and completed several questionnaires (e.g., Yale Food Addiction Scale (YFAS), Brief Resilience Scale (BRS), Early Traumatic Inventory (ETI)). Freesurfer 6 was applied to generate the morphometry of brain regions. A multivariate pattern analysis was used to derive brain morphometry patterns associated with food addiction. General linear modeling and mediation analyses were conducted to examine the effects of ELA and resilience on food addiction in individuals with obesity. Statistical significance was determined at a level of *p* < 0.05. High levels of ELA showed a strong association between reward control brain signatures and food addiction (*p* = 0.03). Resilience positively mediated the effect of ELA on food addiction (*B* = 0.02, *p* = 0.038)**.** Our findings suggest that food addiction is associated with brain signatures in motivation and reward processing regions indicative of dopaminergic dysregulation and inhibition of cognitive control regions. These mechanistic variabilities along with early life adversity suggest increased vulnerability to develop food addiction and obesity in adulthood, which can buffer by the neuroprotective effects of resilience, highlighting the value of incorporating cognitive appraisal into obesity therapeutic regimens.

## Introduction

The national obesity epidemic has increasingly become a public health crisis as obesity-related health complications now account for 61% of annual healthcare costs^1–3^. With an average of 2 in 5 U.S. adults now having obesity, research into environmental factors that increase the risk of obesity, such as early life adversity (ELA), have gained traction to improve treatment outcomes^4^. Early life adversity describes the childhood or adolescent exposure to unfavorable environmental circumstances, such as physical and emotional abuse, trauma, neglect, or family discord^5^.

Research into the links between ELA and obesity remain in its nascent stages although a strong association has been shown in individuals who underwent bariatric surgery^6^. Compared to normative samples, bariatric surgery candidates reported 2–3 times higher rates of childhood maltreatment and an increased risk for psychopathology independent of age, race, and sex^7^. Several explanations have been offered for this relationship including stress-exacerbation, inflammation, even metabolic disturbances and altered eating patterns such as food addiction^8^.

With over 14% of adults and 12% of children today meeting the diagnostic DSM criteria for a substance use disorder, significant controversy exists on whether food addiction should be included in this construct in an effort to help combat this growing prevalence^9,10^. Food addiction is measured by the presence of diagnostic indicators of substance abuse disorder symptoms in the context of ultra-processed/hyperpalatable food intake primarily for pleasure beyond homeostatic needs^11,12^. While binge eating disorder, food addiction, and bulimia nervosa share several overlapping etiologies, recent studies distinguish food addiction from other pathologies given that it may exist in isolation in individuals without eating disorders or obesity^13^. The Yale Food Addiction Scale (YFAS) 2.0 is currently the validated measure to categorize the severity of clinically significant distress/impairment in alignment with the updated DSM-5^14,15^. With the updated classification of substance-use disorders and food addiction, the YFAS 2.0 demonstrated good internal consistency (α = 0.92), discriminative validity, and incremental validity^14,15^.

Although the brain signatures of “reward control” and “reward processing” have overlapping brain regions, these tend to be broad umbrella terms widely used across studies when referring to the reward system. The “reward processing” signature refers to the core regions directly involved in the dopaminergic regulation of inciting stimuli, including the nucleus accumbens, caudate nucleus, and insula^6^. The “reward control” signature refers to the extended reward network containing regions that generally influence/control the core reward system^16^. Compared to those with no food addiction, structural and resting-state neuroimaging studies in individuals with high BMI and food addiction showed lower grey matter volume and lower connectivity in the inferior frontal gyrus, a region involved in response inhibition and appetite control^17,18^. In addition, compared to healthy controls, patients with binge eating disorder and bulimia nervosa showed distinct morphometry in the reward regions, such as decreased grey matter volume in the dorsal striatum/caudate nucleus^19^. While binge eating disorder and bulimia nervosa may share related underlying mechanisms with food addiction, it is important to note that not all individuals with food addiction meet criteria for these disorders. These dysregulations in the mesolimbic reward pathway suggest either an overall deficiency of dopamine or the decreased sensitivity of dopamine (D2) receptors^20,21^. This hypodopaminergic state along with decreased inhibitory control of regions involved in emotional regulation creates a compensatory drive for an increased frequency of reward-seeking behavior, such as the overconsumption of hyperpalatable foods, in order to achieve the same level of satisfaction^22^.

Past literature has used magnetic resonance imaging (MRI) as a tool to explore the neurobiological mechanisms by which early life adversity (ELA) could impact hedonic eating behavior. One explanation suggests that the significant repeated stressors that individuals with ELA experience in their life causes significant increases in glucocorticoid (cortisol) levels^23,24^. Repeated fluctuations in cortisol levels often lead to dysregulation of the hypothalamic-pituitary axis which has been associated with cortical atrophy^23^. In line with this explanation, individuals exposed to ELA show brain volume reductions in areas involved in reward responses and decision making such as the dorsolateral prefrontal cortex, orbitofrontal cortex, and the medial prefrontal cortex^25–27^. The reductions in cortical volume in these regions could decrease the amount of dopamine that is available to mediate reward-seeking behavior causing food addiction^28^. Therefore, due to ELA’s role in promoting addictive behaviors and impairing decision making, it is hypothesized that ELA’s stressful role could spur individuals to use food as a means of comfort and security thus increasing the risk of obesity and food addiction^29,30^.

Studies have also investigated the role of emotional resilience and its association with obesity and ELA^31,32^. Among children at high risk for behavioral problems, high scores on social resilience questionnaires, which measured components of self-esteem and effective stress-coping strategies, attenuated the influence of early adversity in the academic setting^33^. Additionally, in individuals with both high adversity and high resilience, adversity load decreased fMRI activity in the mesolimbic reward system, but resilience improved functional coupling in this system, namely between the ventral striatum, ventral tegmental area, and hippocampus^34^. This suggests that resilience might serve as a protective factor against the adverse brain changes usually seen in individuals with ELA^34^. However, the precise neurobiological mechanisms on how ELA and resilience interact in the context of obesity and food addiction remain fairly unknown.

In this cross sectional study, we aim to determine the role of ELA and resilience in characteristic brain signatures associated with food addiction in order to test the following hypotheses: (1) Food addiction is associated with alterations in brain morphometry in reward processing and cognitive control regions (2) Early life adversity and resilience moderate the relationship between brain morphometry and food addiction (3) Resilience buffers the association between early life adversity and food addiction in individuals with high BMI.

## Materials and methods

### Participant selection and study design

**Figure 1:**
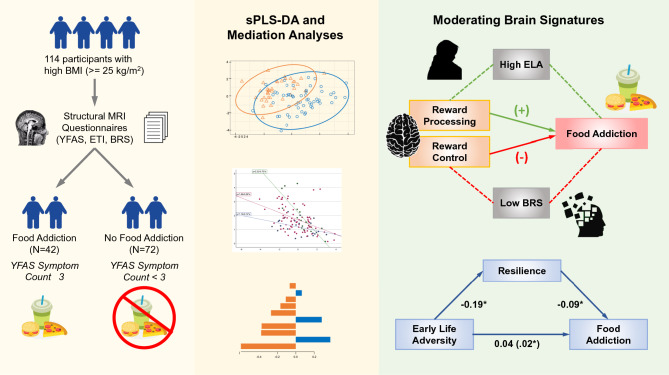
Graphical Abstract.

Figure 1 provides a visual overview of the study design and conducted analyses. A cohort of 114 participants (male: N = 34, female: N = 80) between the ages of 18 and 60 years was recruited through the University of California, Los Angeles with local community advertisements similar to previous studies^35–37^. All participants had a high BMI (> = 25 kg/m^2^). Similar to previous studies, participants were excluded for the following: significant medical/neurological conditions, past/present psychiatric illnesses, pregnancy or breastfeeding, substance use, weight loss/abdominal surgery, tobacco dependence (half a pack or more daily), extreme strenuous exercise (> 8 h of continuous exercise per week), and metal implants^38–40^. Participants taking medications that interfere with the central nervous system or regular use of analgesic drugs were excluded^39^. Due of the effect of handedness on brain signatures and brain function related to laterality, only right-handed participants were included^41^. Only premenopausal females and those who were scanned during the follicular phase of their menstrual cycle according to self-report measures were included, since female sex hormones such as estrogen have been known to affect brain structure and function^42^. Participants with hypertension, diabetes, or metabolic syndrome were excluded to minimize confounding effects. Additionally, participants were screened for psychiatric and eating disorders using the MINI Mental State Examination (MMSE) and an in-depth interview. Those who endorsed having an eating disorder, bulimia nervosa, or incidence of binge eating were excluded from the study. No participants exceeded 400lbs due to magnetic resonance imaging (MRI) scanning weight limits. All procedures were in compliance with institutional guidelines and were approved by the Institutional Review Board at UCLA’s Office of Protection for Research Subjects (IRB#s 16-000281, 16-000187, 20-002326). All participants provided written informed consent.

### Questionnaires

To assess eating behaviors, early life adversity, and level of resilience, each participant was asked to fill out the Yale Food Addiction Scale (YFAS, food addiction), Early Traumatic Inventory (ETI, ELA) and Brief Resilience Scale (BRS, resilience) questionnaires.

Data for eating behaviors was assessed using the Yale Food Addiction Scale (YFAS), a 25-item questionnaire that measures food addiction to highly palatable foods (high sugar/high fat foods) by assessing signs of substance-dependence symptoms as outlined in the DSM-IV^43^. This scale measures several aspects of food addiction behavior: food dependence, withdrawal, tolerance, continued use despite problems, time spent eating, loss of control, inability to cut down, and clinically significant impairment^44,45^. For discriminative analysis, individuals were grouped by food addiction (FA) was defined as having a YFAS symptom count ≥ 3 with clinically significant impairment or distress (N = 42 with FA, N = 72 with no FA). Clinically significant impairment or distress was defined as having at least one positive response to the following two questions in the YFAS questionnaire: “My behavior with respect to food and eating causes significant distress” and “I experience significant problems in my ability to function effectively (daily routine, job/school, social activities, family activities, health difficulties) because of food and eating,” similar to previously published works^46,47^. A higher score on this questionnaire indicates higher binge eating scores or binge eating days, while a lower score indicates lower incidents of food overconsumption^48^. The YFAS has displayed good internal reliability (Cronbach’s α = 0.86) in a previous research study based on a 353-respondent survey^49^ and this was similar for the current sample (Cronbach’s α = 0.85).

Early life adversity (ELA) was measured using the Early Traumatic Inventory-Self Report (ETI-SR), a 27-item questionnaire that assesses the histories of childhood traumatic and adverse life events that occurred before the age of 18 years old and covers four domains: general trauma (11 items), physical punishment (5 items), emotional abuse (5 items), and sexual abuse (6 items)^50^. Questions in this questionnaire included death of a parent or loved one, discordant relationships, physical constraint or confinement, and forced coercion into sexual acts against one’s will^50^. The ETI-SR instrument was chosen due to its high internal consistency (Cronbach’s α = 0.95, current sample α = 0.96), ease of administration, and ability to measure ELAs across multiple domains in a time efficient manner^50^.

The Brief Resilience Scale (BRS), is a 6-item unidimensional survey solely focused on the ability of an individual to recover from stress and adversity, and it is most commonly used in clinical practice to evaluate treatment of depression and anxiety^51,52^. The total BRS score ranges from 0 to 30, with higher scores indicating higher resilience in terms of bounce-back ability^53^. The BRS has been used in research studies in cancer patients, vocational rehabilitation service recipients, and the general adult population, and has demonstrated moderate internal consistency (Cronbach’s α = 0.71)^54–56^ and for the current sample internal consistency was quite good at (Cronbach’s α = 0.80).

### Imaging acquisition and processing

#### Brain MRI: acquisition

Whole brain structural images were acquired using a 3.0T Siemens Prisma MRI scanner (Siemens, Erlangen, Germany). Detailed information on the standardized acquisition protocols, quality control measures, and image preprocessing are provided in previously published studies^57–62^.

#### Structural MRI acquisition

High resolution T1-weighted images were acquired: echo time/repetition time (TE/TR) =3.26 ms/2200 ms, field of view =220 × 220 mm, slice thickness=1 mm, 176 slices, 256 × 256 voxel matrix, and voxel size = 0.86 × 0.86 × 1 mm.

#### Brain MRI: structural image parcellation

T1-image segmentation and cortical and subcortical regional parcellation were conducted using the Destrieux, the Harvard–Oxford subcortical atlas and the Ascending Arousal Network atlases in Freesurfer^63–65^. For each hemisphere, this parcellation results in the labeling of 74 cortical structures in addition to 7 subcortical structures including the cerebellum, and 14 brainstem nuclei for a total of 178 parcellations. After parcellation, the volume, cortical thickness, surface area and mean curvature was computed for each region. These subject-specific morphometric indices were residualized using the total intracranial volume of each subject.

### Statistical analyses

To determine whether there were brain signatures that could discriminate low and high FA groups, we employed sparse partial least squares—discriminate analysis (sPLS-DA). sPLS-DA is a latent variable approach that operates by using a supervised framework forming linear combinations of the predictors (i.e., regional volumes, cortical thickness, surface area, or mean curvature) based on class membership (i.e., high/Low FA) and reduces the dimensionality of the data by finding a set of orthogonal components each comprised by a selected set of features or variables^66,67^. These components are referred to as brain signatures. Each variable comprising a brain signature has an associated “loading,” which is a measure of the relative importance of the variables for the discrimination into two groups^66^. The values or scores for each participant each component/signature are plotted in two-dimensional space to visualize the discrimination in a sample plots as well as can be extracted for downstream analysis. Our data set was split (80/20) into a training and test set. The predictive accuracy of the signatures derived from the training set were assessed in the test data by calculating the balanced error rate (BER), the average proportion of wrongly classified samples in each class, weighted by the number of samples in each class. Balanced accuracy or Area under the curve (AUC), the average of the sensitivity and the specificity, is calculated as 1-BER. Additional details about the use of sPLS-DA in discrimination analyses can be found in our previous study^59,61,68,69^.

Individual subject scores on the derived signatures were extracted and entered into subsequent analyses. Independent t-test and Cohen’s effect size d were used quantify the magnitude of group differences in the derived brain signatures and the study variables. We performed a moderator analysis to determine if participant levels of ELA or BRS might influence or moderate the association between the brain signatures and food addiction. General linear modeling was applied to test the moderating effect of ETI and BRS on the association between the two derived brain signatures and FA, as determined by the total YFAS Symptom Count. In this model, we specified main effects and two-way interactions for ETI, BRS, reward processing signature, and the reward control signature. BMI was included as a main effect. A significant interaction involving the moderators (ELA or BRS) suggests the association between the brain signature and food addition scores depends on the level of the moderator. To aid in interpretation, of the moderator effects, parameter estimates were displayed graphically at low, moderate, and high levels of each moderator variable (− 1 SD, mean, + 1SD).

To examine the extent to which the effects of early life adversity on FA were mediated by resilience we applied a mediation model using simultaneous linear regression equations with maximum likelihood estimation in AMOS 6.0 with standard errors and bootstrapping based on 95% confidence intervals for path regression estimates generated with calculated based on the normal distribution. In this model, ETI served as the independent variable, BRS, the mediating variable, and food addiction was the DV, as measured by the total YFAS count. BMI was included as a covariate. For the indirect effect a confidence interval that do not include zero suggests a mediation effect is present. All variables were mean centered for mediator and moderator analyses.

## Results

### Participant characteristics and results of questionnaires

Participant characteristics and results of questionnaires are summarized in Table 1.

**Table 1:** Study demographic and behavioral characteristics.

	No food addiction (N = 72)		Food Addiction (N = 42)		t	*P*	Cohen’s D
	Mean	SD	Mean	SD			
Age	33.24	10.92	30.33	9.35	1.44	0.150	0.28
BMI	30.38	4.11	32.93	5.22	-2.89	0.005	-0.56
ETI total score	4.08	4.51	6.24	4.42	-2.48	0.015	-0.48
BRS score	23.61	4.28	21.93	5.02	1.88	0.060	0.37

Means and standard deviations (SD) are reported for normally distributed data.

BMI, Body Mass Index; BRS, Brief Resilience Scale; ETI, Early Traumatic Inventory Questionnaire; SD, Standard Deviation; *p*-value significant < 0.05.

The study cohort included 114 participants and consisted of low FA (N = 72, mean BMI = 30.38, SD = 4.11) and high FA (N = 42, mean BMI = 32.93, SD = 5.22). The mean age of the low FA cohort (mean = 33.24, SD = 10.92) was similar to the high FA group (mean = 30.33, SD = 9.35). Participants with low FA (N = 72) show higher resilience (*p* = 0.015, Cohen’s d = 0.37), lower levels of ELA (Cohen’s d = − 0.48). There were no significant differences in age or distribution of sex by FA subtype (*p* = 0.150).

### Associations of brain networks with food addiction

**Figure 2:**
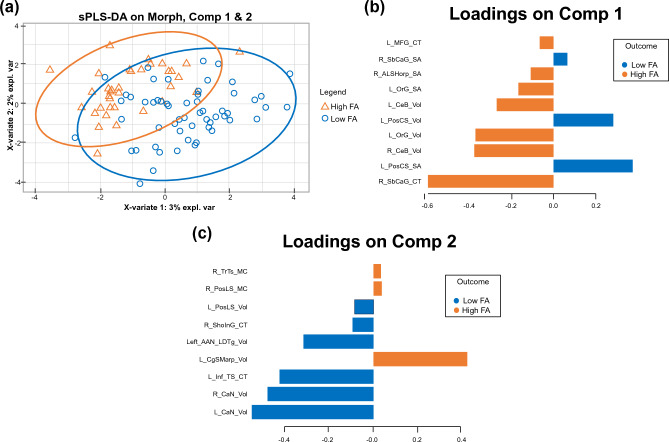
**(A)** Brain Signatures Associated with Food Addiction. Abbreviations: sPLS-DA, sparse partial least squares – discriminate analysis. (**B)** Regions that Exert Cognitive Control over Reward Processing. Orange bars indicate higher values in individuals with food addiction and blue bars indicate higher values in individuals with no food addiction. Abbreviations: ALSHorp, Horizontal Ramus of the Anterior Segment of the Lateral Sulcus; CeB, Cerebellum Cortex; MFG, Middle Frontal Gyrus; OrG, Orbital Gyri; PosCS, Postcentral Sulcus; SbCaG, Subgenual Anterior Cingulate. (**C**) Reward Processing Regions. Orange bars indicate higher values in individuals with food addiction and blue bars indicate higher values in individuals with no food addiction. Abbreviations: CaN, Caudate Nucleus; CgSMarp, Marginal branch of the cingulate sulcus; InfTS, Inferior Temporal Sulcus; LDTg, Laterodorsal Tegmental Nucleus; PosLS, Posterior Ramus of the Temporal Sulcus; SholnG, Short Insular Gyri; TrTS, Transverse Temporal Sulcus.

The sPLS-DA revealed two brain signatures that discriminated the FA groups (Fig. 2A).

The brain features selected on the first component were regions known to exert control of reward processing, including the subgenual anterior cingulate (sACC), orbitofrontal and dorsolateral prefrontal cortex and anterior insula. As shown in the loading plot (Fig. 2B), individuals with high FA had greater cortical thickness of the sACC and greater surface area and volume of the orbital gyrus, while individuals with low FA had greater surface area and volume of the posterior central sulcus. In general, scores this Reward Control signature were higher in the low FA compared to the high FA group, t(112) = 3.65, *p* = 0.0004, Cohen’s d = 1.00.

The second brain signature was comprised of brain regions involved in reward processing regions. As shown in Fig. 2C, participants with FA had greater volume of the caudate nucleus, greater cortical thickness of the short insular gyrus, and greater volume of the laterodorsal tegmentum nucleus. Overall, participants with high FA had higher scores on this Reward Processing signature, t(112) = − 3.0, p = 0.003, Cohen’s d = 0.78.

Together these two signatures discriminated the FA groups with a balance error rate = 41.4% and area under the curve of 58.6%, indicating that brain morphometry does not fully account for food addiction.

### Impact of early life adversity on food addiction

**Figure 3:**
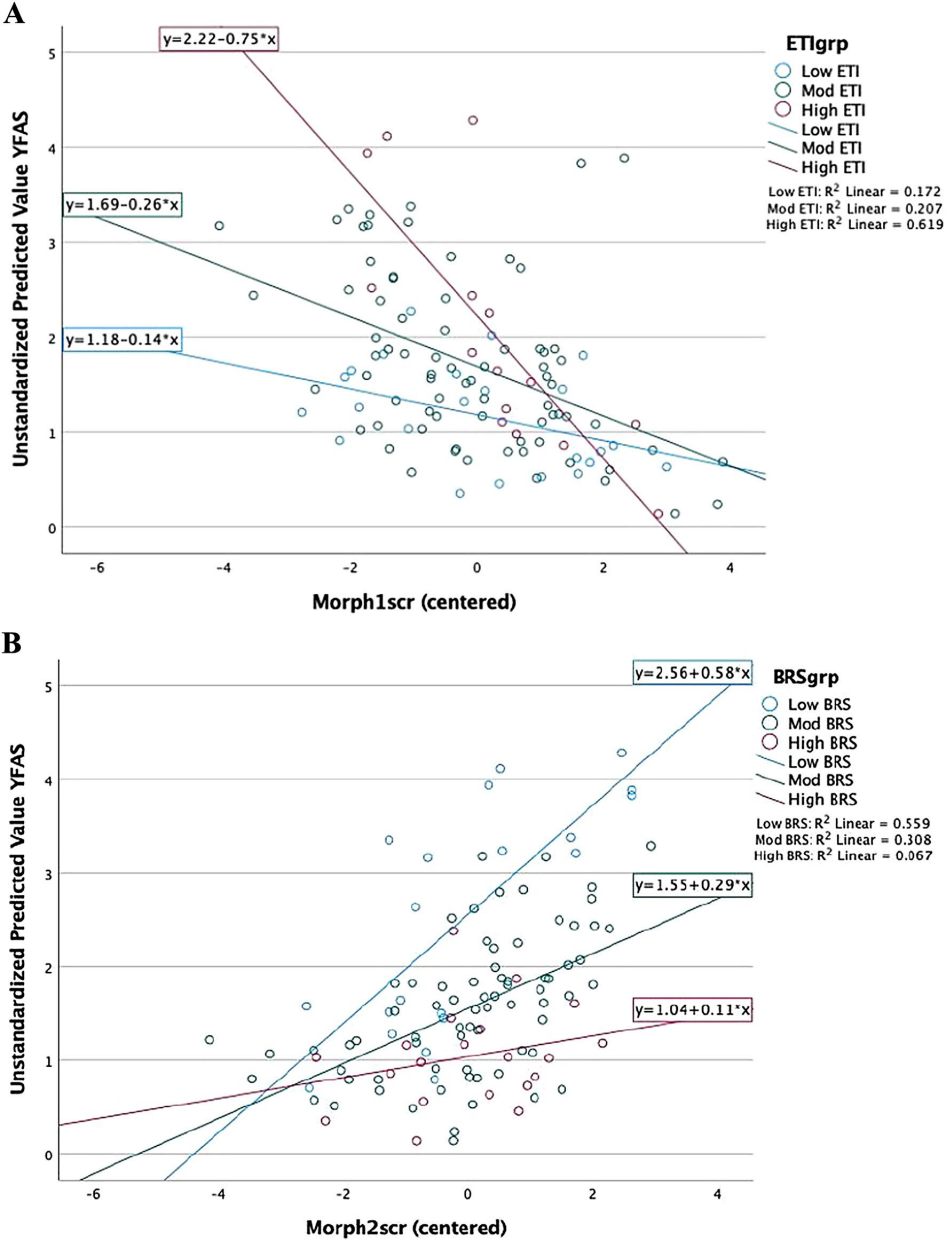
Moderating effects of early life adversity and resilience on the association between brain signatures and food addiction. Abbreviations: BRS, Brief Resilience Scale; ELA, Early Life Adversity; FA, Food Addiction; *p*-significant < 0.05.

Across all participants, higher scores on the Reward Control signature was associated with lower food addiction, Beta (SE) = − 0.34 (0.06), t = − 4.00, *p* = 0.4.2×e−7. ELA significantly moderated this association between the Reward Control signature and FA, Beta (SE) = − 0.03 (0.014), t = − 2.14, *p* = 0.03. As shown in Fig. 3A, participants with the highest levels of ELA showed the strongest negative association between the Reward Control brain signature and FA scores (see Table 2).

**Table 2:** Parameter estimates from the Moderator Analysis.

Parameter	UnstdB	S.E.	t	Sig	95% Confidence interval		Partial Eta squared
					Lower bound	Upper bound	
BMI	.066	.021	3.146	.002	.025	.108	.089
ETI	.056	.022	2.498	.014	.011	.100	.058
BRS	−.086	.021	−3.999	< .001	−.129	−.043	.137
Reward Control (RC)	−.339	.063	−5.411	< .001	−.463	−.214	.225
Reward Processing (RP)	.273	.071	3.842	< .001	.132	.414	.128
ETI*BRS	.004	.005	.882	.380	−.005	.013	.008
ETI*RC	−.031	.014	−2.141	.035	−.060	−.002	.043
ETI*RP	−.009	.017	−.558	.578	−.042	.024	.003
BRs*RC	.009	.013	.698	.487	−.017	.036	.005
BRS*RP	−.030	.015	−2.054	.043	−.059	−.001	.040
RC* cRP	.064	.054	1.192	.236	−.043	.172	.014

*Dependent Variable to assess FA: YFAS_SymptomCount.

Abbreviations: Body Mass Index (BMI); Brief Resilience Scale (BRS); Early Traumatic Inventory (ETI); Reward Control (RC); Reward Processing (RP).

### Impact of resilience on the association of early life adversity and food addiction

Overall participants’ higher scores on the Reward Processing signature were positively associated with lower FA, Beta(SE) = 0.27 (0.07), t = 3.84, *p* = 0.002. BRS significantly moderated this association between the Reward Processing signature and FA, Beta (SE) = − 0.03(0.015), t = − 2.05, *p* = 0.043. As shown in Fig. 3B, participants with the lowest levels of resilience showed the strongest positive relationship between the Reward Processing brain signature and FA scores (See Table 2).

### Resilience mediated the effect of early life adversity on food addiction

**Figure 4:**
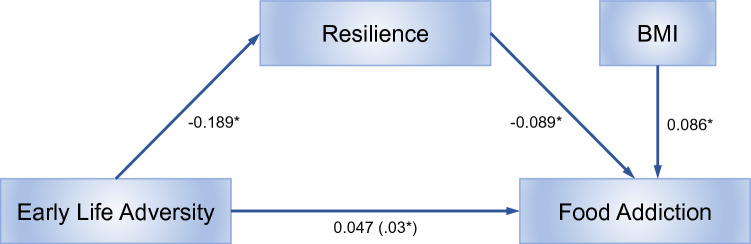
Resilience mediates the relationship between early life adversity and food addiction. Abbreviations: BMI, Body Mass Index; ELA, Early Life Adversity; FA, Food Addiction; *p*-value significant < 0.05; YFAS, Yale Food Addiction Scale.

Results from the mediation analysis are provided in Table 3 and Fig. 4*.* After controlling for BMI, resilience significantly mediated the effect of ELA on FA, B = 0.02, 95% CI 0.001,0.043). The proportion of the effect of ELA on FA mediated by resilience was 36%.

**Table 3:** Regression weights in the model.

Path	UnstdB	SE	p	z	95% Confidence interval	
					Lower bound	Upper bound
ETI→BRS	− 0.189	0.097	0.043	-2.02	− 0.42	− 0.004
ETI→YFAS	0.047	0.027	0.045	1.96	0.002	0.112
BRS→YFAS	− 0.089	0.027	0.012	-3.75	− 0.14	− 0.036
BMI→YFAS	0.086	0.024	0.009	3.75	0.034	0.132
IOM	0.017	0.010			0.001	0.043

BMI, Body Mass Index; BRS, Brief Resilience Scale; CI, Confidence Interval; ETI, Early Traumatic Inventory; IOM, Indirect effect of Moderator, SE, Standard Error; YFAS, Yale Food Addiction Scale.

## Discussion

The goal of the present study was to investigate the interactions between ELA, resilience, alterations in the brain’s reward network, and FA in individuals with high BMI. Consistent with the substance use disorder literature, individuals with high levels of FA exhibited increased cortical thickness in the sACC and decreased volume in the caudate nucleus^70^. While both substance use disorders and FA contain links to dopaminergic dysregulation in reward circuits as seen from our results, previous studies do state the important consideration that substance use disorders display more robust neuroadaptive effects with higher “addictivity,” withdrawal, and tolerance^71,72^. Furthermore, ELA moderated the association between FA and a reward control brain signature while resilience moderated the relationship between reward processing brain signatures and FA. These results support the hypothesis that ELA events during childhood may alter structural morphometry of brain regions in the extended reward network and increase vulnerability for FA and obesity in adulthood. While our findings corroborate the previously noted increased cortical volume/thickness in the brainstem and extended reward network regions between individuals with and without FA, this is one of the first studies to investigate the role of ELA on brain networks, high BMI, and FA while accounting for the mediating role of resilience in these associations^18,73^.

### Brain signatures associated with food addiction

**Figure 5:**
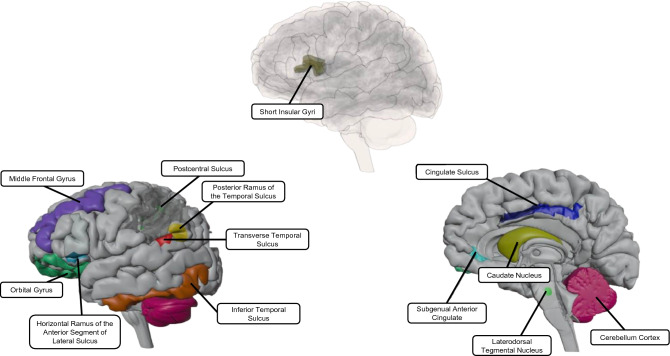
Key Regions in the Extended Reward Network. Abbreviations ALSHorp, Horizontal Ramus of the Anterior Segment of the Lateral Sulcus; CaN, Caudate Nucleus; CeB, Cerebellum Cortex; CgSMarp, Marginal branch of the cingulate sulcus; InfTS, Inferior Temporal Sulcus; LDTg, Laterodorsal Tegmental Nucleus; MFG, Middle Frontal Gyrus; OrG, Orbital Gyri; PosCS, Postcentral Sulcus; PosLS, Posterior Ramus of the Temporal Sulcus; SbCaG, Subgenual Anterior Cingulate SholnG, Short Insular Gyri; TrTS, Transverse Temporal Sulcus.

Two notable brain signatures were seen in individuals with FA: a reward processing brain signature and a reward control brain signature. Regions of the extended reward network involved in these brain signatures are depicted in Fig. 5. Higher cortical thickness and surface area in areas of reward processing, such as the caudate nucleus, was primarily seen in individuals with low FA. This suggests that the converse could also be true: namely, individuals with FA might display lower cortical thickness in regions of reward processing. Along with other nearby regions of the basal ganglia, the caudate nucleus is involved in reward-dependent motivation and incentive salience of pleasant stimuli^74–76^. Due to the caudate’s involvement in the reward circuit, lowered cortical thickness in this region could suggest a lowered availability of dopamine (D2) receptors is responsible for sending dopaminergic projections to the nucleus accumbens, another key reward region^77–79^. According to the reward deficiency hypothesis, this decreased receptor availability leaves individuals in a perpetually hypodopaminergic state causing them to seek frequent, and larger increments of reward stimulation in a compensatory mechanism to achieve the same euphoric effect^80,81^. This potent reward trigger could translate to the consumption of high sugar/high fat foods in individuals with food addiction causing cravings that may override internal satiety cues by modulating the relative incentive salience of these palatable foods^18,82^. Thus, the reward processing brain signature observed may corroborate our initial hypothesis since the lowered cortical thickness in the caudate nucleus could be associated with the dopaminergic dysregulation often seen in those with FA.

The reward control brain signature indicated greater cortical thickness and surface area in regions such as the subgenual anterior cingulate (sACC) and the orbital gyrus in individuals with food addiction. Although few studies have explored the functional importance of the sACC, notable reductions of the cortex volume have been observed in patients with major depressive and bipolar disorder^83,84^. Due its connections with the amygdala, ventral striatum, and autonomic centers, the sACC may contribute to affect by sustaining autonomic arousal in anticipation of incoming stimuli^85^. In the case of depression, lowered volume of the sACC could result in an unnecessary level of sustained arousal in response to negative stimuli, resulting in a domination of negative emotions for the patient^84^. In the case of FA, increased cortical thickness of the sACC may play a role in elevating autonomic arousal to positive stimuli such as palatable food thus increasing expectation and anticipation beyond baseline^86,87^. This could result in greater cravings since the overwhelming anticipation of reward could inhibit cognitive control regions and an increasing susceptibility for compulsive overeating behaviors^88^.

### Moderating effects of ELA and resilience on food addiction

Based on our findings, both the reward control and reward processing brain signature do not fully account for FA pathology since the area under the curve as determined by Fig. 2A remained below 60%. This indicates the possible presence of moderating factors, namely ELA and resilience, that could further explain the association between these brain signatures and higher FA scores.

Compared to individuals with lower levels of ELA, those with higher levels of ELA showed a stronger association between regions of reward control and FA scores. Early deprivation and trauma, common in ELA, may disrupt maturation of reward circuits and dampen behavioral and neural sensitivity to pleasurable stimuli^89–91^. These alterations could also impact functioning in specific neurocognitive regions, such as the anterior cingulate cortex (ACC), that could influence decision-making, cognitive inhibition, emotion, and motivation^92^. Lowered cortical thickness in the ACC potentially contributes to decisions that favor enhanced reactivity to threat and provides enhanced motivation for reward-stimulating behaviors, such as compulsive overeating^93^. This could explain the increased frequency of reliance on palatable foods seen in individuals with early life adversity as a compensatory response for the trauma-induced reduced reward responsivity^94^. Thus, ELA may contribute to disruptions in the topology of these brain regions and increase vulnerability to develop FA, relative to changes seen in FA alone.

Our findings also demonstrated resilience as a moderating factor since individuals with low resilience had a stronger association between the reward processing brain signature and FA scores. As mentioned earlier, alterations in reward processing, such as in the caudate nucleus, could be attributed to compromised dopaminergic signaling as a result decreased receptor availability and cause hedonic eating behaviors. Although research surrounding the neural correlates of resilience remain scarce, studies show that modulation of dopaminergic neurons confer resilience to chronic stress^95^. Alcohol and drug addiction studies show that higher striatal dopamine D2 receptor availability in the striatum might promote resilience to alcohol use disorders, higher positive emotionality, and greater resistance to the reinforcing effects of stimulants^96–98^. In addition, individuals with PTSD, a marker of low resilience, have dysregulation of dopamine in the mesolimbic pathway which contributes to their inattention to incoming stimuli^99^. Therefore, it can be speculated that low resilience groups have decreased availability of D2 receptors and impaired inhibitory control of dopaminergic neurons which is evidenced by lower cortical volumes of reward processing regions and higher FA scores^71,100–102^.

### Mediating effect of resilience on ELA and degree of food addiction

Our results indicate that resilience can act as a buffer against the effects of deleterious effects of ELA which lead to FA by enacting neuroprotective changes to brain morphology. While this study measured resilience through a behavioral questionnaire, one possible explanation is that resilience allows an individual to increasingly recruit regions of the frontoparietal network as a compensatory mechanism for the decreased cortical volume of reward-related decision-making regions caused by early chronic stress^103^. This allows for the modulation of cognitive control and reward processing areas in response to both positive and negative emotional cues^98,104^. By utilizing cognitive appraisal as a strategy to combat impulsive emotional responses, the incidence of pathologies such as FA could be diminished.

### Limitations and future directions

While the ETI serves as a cost-effective tool to succinctly gather information about early life adversity, there is an inherent risk of recall bias since participants may not be able to accurately recall all aspects of their childhood. Additionally, longitudinal studies are needed to determine if FA causes rewiring in brain architecture and/or if ELA is the primary driver in shaping brain networks and predisposing an individual to maladaptive eating behaviors. Future mechanistic studies should explore the brain morphometric changes in how ELA modulates the pathology of food addiction with a focus on sex differences. The integration of gut metabolites and functional connectivity differences could also provide a more comprehensive understanding of the relationships between oxidative stress, ELA, and associated neural changes.

### Clinical implications and conclusions

When viewed together with previous reports, our results show that ELA results in morphometric changes to the reward circuit and increases susceptibility to food addiction in individuals with high BMI^6,27,105^. However, resilience has a neuroprotective role in mitigating the adverse structural effects of ELA contributing to hedonic eating behaviors. These findings illustrate the importance of comprehensively exploring one’s developmental history, including ELA, when determining therapeutic avenues for food addiction and obesity later in life. Associated behaviors such as depression, emotional dysregulation, and impulsivity have similar brain morphology and have strong associations with individuals with FA^106,107^. These observed mechanistic changes also help understand the clinical significance of individualized treatment efforts for individuals with FA that boost protective factors, such as social support and resilience.

## Data Availability

The datasets generated and/or analysed during the current study are not publicly available due to this being an ongoing clinical study but are available from the corresponding author on reasonable request.
